# Difficulties in diagnosis and treatment of severe secondary Raynaud’s phenomenon in a Cameroonian woman: a case report

**DOI:** 10.1186/s13256-016-1142-x

**Published:** 2016-12-20

**Authors:** Valirie Ndip Agbor, Tsi Njim, Leopold Ndemnge Aminde

**Affiliations:** 1 Ibal Sub-Divisional Hospital, Oku, North West Region Cameroon; 2 Centre for Tropical Medicine and Global Health, Nuffield Department of Medicine, University of Oxford, Oxfordshire, UK; 3 Clinical Research Education, Networking and Consultancy (CRENC), Douala, Littoral Cameroon; 4 School of Public Health, Faculty of Medicine & Biomedical Sciences, University of Queensland, Brisbane, Australia

**Keywords:** Secondary Raynaud’s phenomenon, Connective tissue disease, Cameroon

## Abstract

**Background:**

Raynaud’s phenomenon is a microvascular disorder that results in exaggerated vasoconstriction over vasodilatation secondary to an alteration in autonomic control. Though benign, it can result in severe ulceration and ultimately gangrene associated with disfiguration and permanent deformity. We present a case of severe secondary Raynaud’s phenomenon in a black-African patient from a resource-limited setting, with focus on the difficulties encountered in the diagnosis and treatment.

**Case presentation:**

A 43-year-old female Cameroonian farmer with a 7-year history of episodic paresthesia in her fingers and toes (when exposed to cold) presented to our emergency department with severe pain, ulceration, and “darkening” of her fingertips over a period of 2 days. An examination revealed bilateral ulceration and dry gangrene of her fingers and toes, based on which a diagnosis of secondary Raynaud’s phenomenon due to a connective tissue disease was proposed. Results of paraclinical investigations were normal. Lifestyle modification along with a calcium channel blocker and phosphodiesterase type 5 inhibitor provided significant relief.

**Conclusions:**

An early diagnosis and knowledge on appropriate treatment of Raynaud’s phenomenon is of vital importance to prevent permanent tissue damage and disability. Relying on biphasic color change for the diagnosis of Raynaud’s phenomenon in black Africans can be potentially misleading.

## Background

Raynaud’s phenomenon (RP) is a microvascular disorder generally involving the digits and other extremities such as the nose, ears, and nipples [1]. This phenomenon was first described in 1862 by the French physician Maurice Raynaud [2]. In extreme severity it can lead to ulceration and gangrene of the affected extremities, resulting in disfiguration and permanent disability. Herein, we describe a case of severe secondary RP in a black African woman from a resource-limited setting, and we discuss the difficulties encountered in the diagnosis and management.

## Case presentation

**Fig. 1:**
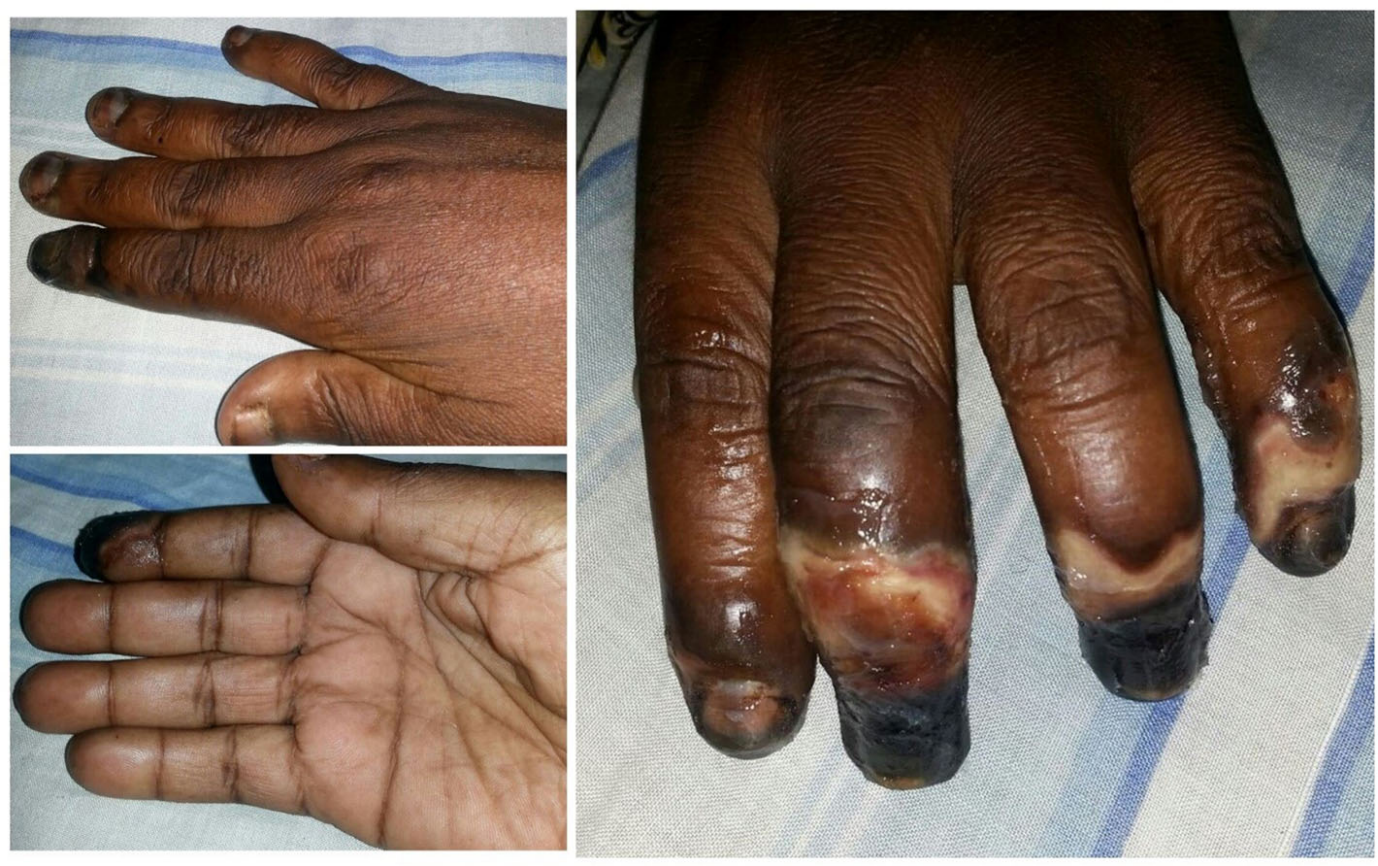
Dorsal and palmar view of ulceration and gangrene of the distal third of the right index finger (*left*), and the distal third fingers of the left hand (*right*)

**Fig. 2:**
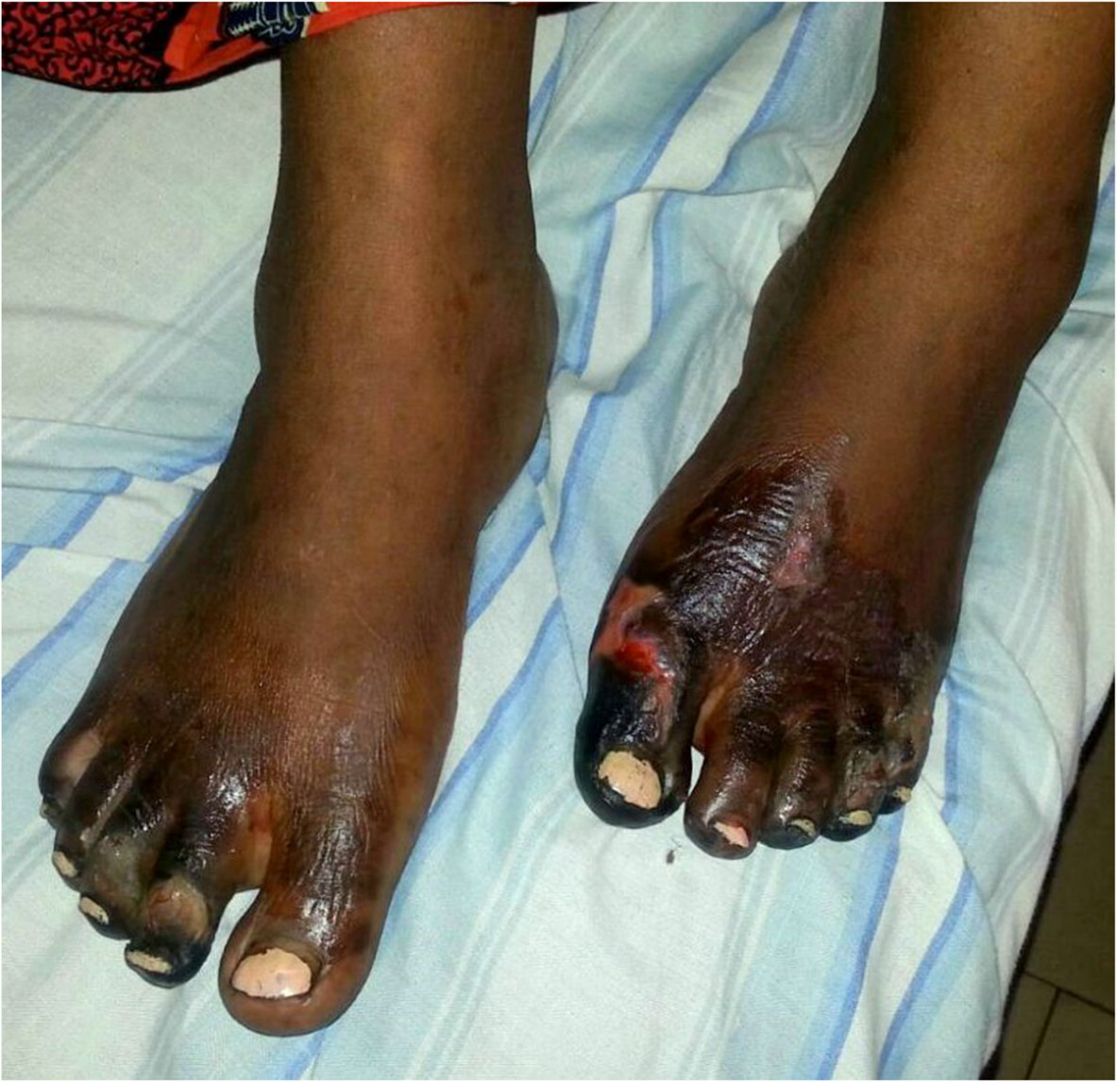
Symmetric affection with ulceration and gangrene of the toes

A 43-year-old female Cameroonian farmer presented to our emergency department with pain, ulceration, and “darkening” of her fingers and feet of 2 days’ duration. The pain was mild in intensity at the onset, then progressively worsened over 2 days. She took self-prescribed doses of diclofenac that temporarily relieved the pain. Resurgence of the pain with the onset of ulceration motivated her present consultation. Her past history was remarkable for episodic “pins and needles” sensation of the fingers aggravated by cold (mostly cold weather and immersion of hands in cold water). She had bilateral knee and elbow joint pain, but no color changes of her digits prior to the onset of ulceration and gangrene. On physical examination, she was anxious and in painful distress with a blood pressure of 156/94 mmHg, a pulse rate of 94 beats per minute, a respiratory rate of 28 cycles per minute, and a temperature of 37.4 °C. There were dry gangrenous lesions affecting the distal third of the middle, ring, and small finger of her left hand and the second finger of her right hand (Fig. 1), and the distal extremity of her feet (Fig. 2). No other cutaneous lesions were observed. The rest of the physical examination was not contributory. A presumptive diagnosis of severe secondary RP due to a connective tissue disease was made.

Her erythrocyte sedimentation rate (ESR), fasting blood sugar, anti-streptolysin O antigen (ASLO) level, human immunodeficiency virus (HIV) serology, urine analysis, and rheumatoid factor concentration were all negative. A Doppler ultrasonography of her peripheral blood vessels was normal. Blood samples for anti-nuclear antibodies (ANA) and anti-topoisomerase (anti-Scl 70) antibodies were collected and preserved to be sent abroad for analysis, though plans fell through due to financial constraints.

We admitted her to our intensive care unit. Non-pharmacological treatment included stopping diclofenac (and other prostaglandin inhibitors); putting on warm clothing; placing her hands and feet in lukewarm water; and avoiding consumption of caffeine-containing products, cold exposure, and smoking (active or passive). Pharmacological measures included nifedipine, 20 mg per os thrice daily; sildenafil, 15 mg per os thrice daily; cloxacillin, 500 mg thrice daily; tramadol, 100 mg start dose given intravenously; and open dressing of her ulcers. Complete analgesia was achieved within 45 minutes and, after 3 days of inpatient management, our patient was stable and referred to a rheumatologist for further management but was lost to follow-up.

## Discussion

Globally, primary RP is fairly common with a prevalence of 3–5% predominantly in cold climates [1]. The prevalence of secondary RP depends on the associated disease, reaching up to 90% in patients with systemic sclerosis [3]. No epidemiologic data on this phenomenon is available in Africa.

The following have been suggested as driving factors in RP: cold (most common), emotional stress, drugs such as beta blockers, prolonged use of digits (like prolonged typing and piano playing), smoking, and the presence of an underlying vascular disease [1]. Cold and farming (associated with prolonged use of digits) were most likely the trigger factors of our patient’s crisis, with possible aggravation by diclofenac, which inhibits prostaglandin synthesis by inhibiting cyclo-oxygenase.

RP results from an exaggeration of vasoconstriction of the pre-arteriolar capillaries over vasodilatation, secondary to an alteration in the neurological control of the vasomotor tone and circulating mediators [4]. The autonomic nervous system overreacts and overproduces endothelin 1 (a potent vasoconstrictor also associated with vascular fibrosis) with respect to prostaglandin E_2_ [PG E_2_ (a vasodilator)], leading to excessive vasoconstriction [5]. This results in the episodic and symmetrical triphasic color change (pallor, cyanosis, and rubor), trophic skin changes, and uncomfortable sensory symptoms in the involved extremities (paresthesia in our case) [6]. On exposure to cold, the distal finger pads initially become pale as a result of vasoconstriction, then cyanosis occurs, secondary to tissue hypoxia, and finally they turn red, as tissue reperfusion occurs [1]. RP has been classified clinically into two types: primary RP, which is mostly benign; and secondary RP, which in 10–20% of cases is associated with an underlying systemic disease [such as Sjören’s syndrome, systemic sclerosis, CREST syndrome (calcinosis, RP, esophageal dysmotility, sclerodactyly, and telangiectasia), systemic lupus erythematosus, or dermatomyositis], or with drugs or extrinsic vascular obstruction [7]. Primary RP is characterized by symmetrical attacks on the digits in the absence of peripheral vascular disease, tissues necrosis, ulceration, or gangrene [2, 8]. Meanwhile, the attacks of secondary RP are associated with more severe asymmetric affection with tissue necrosis, ulceration, and gangrene [2, 8]. Scleroderma syndrome, which can present as CREST syndrome (also called limited systemic sclerosis) and systemic sclerosis (also called diffuse systemic sclerosis) have been associated with severe secondary RP [8]. Our patient, however, had no features suggestive of CREST syndrome. The presence of digital tissue ulceration and gangrene associated with a history of arthralgia in our patient supports the diagnosis of secondary RP due to a systemic disease, but owing to some adversities we could not establish a precise etiology.

The diagnosis of RP in people of black ethnicity can be challenging. Among others, an important hallmark for the diagnosis of RP is the presence of a biphasic color change [1]. This feature is difficult to appreciate in the absence of an attack and in a dark-skinned patient like the index case. Indeed, our patient only presented with pallor of the affected fingers. This raises a potential limitation of this feature as a major diagnostic criterion for RP in black Africans. Although capillaroscopy has a high positive predictive value in detecting the likelihood of a patient having or developing a connective tissue disease [9], this could not be performed due to the limitations of our health facility. In addition, a normal ESR and ASLO and rheumatoid factor concentrations were not indicative of a systemic disease. Furthermore, an analysis of ANA and anti-Scl 70 antibodies could not be done owing to financial constraints and the limited availability of these tests in Cameroon at the time.

There is currently no cure nor gold standard therapy for RP [10] even though many advances have been made as far as pharmacological treatment is concerned [3]. The management of RP depends on whether it is primary or secondary, and it generally involves non-pharmacological and pharmacological measures. Non-pharmacological treatment entails lifestyle modifications such as avoiding cold temperatures, emotional stress, caffeine, smoking, and vasoconstrictive drugs [3]. Lifestyle modification may potentially suffice for the management of primary RP. In cases where lifestyle modification is inadequate, calcium channel blockers (CCBs) or topical nitrates can be added [3].

In secondary RP, pharmacological treatment usually accompanies lifestyle modifications. Iloprost (a prostaglandin analog), sildenafil (a phosphodiesterase type 5 inhibitor), topical nitrates (vasodilators), and bosentan (an endothelin receptor antagonist) could be added to CCBs for patients who experience persistent intense pain, ulceration, and gangrene [3]. Iloprost and sildenafil can reduce the frequency, duration, and severity of attacks, and also improve ulcer healing. Bosentan reduces the incidence of new digital ulcers [3]. In patients with persistent severe ischemia, a surgical consultation for digital sympathectomy is recommended [8]. With lifestyle modification and treatment with nifedipine and sildenafil, signs of ischemia were halted in our patient. Iloprost was not administered, because it was unavailable in local pharmacies.

Unfortunately, our patient presented for treatment with the ultimate complication of her crisis, gangrene, affecting several digits. The only treatment available for gangrene is amputation, resulting in permanent disability.

## Conclusions

We have presented a case of severe secondary RP in a black African woman from a resource-limited setting. Early diagnosis of this condition and appropriate management is pivotal to prevent tissue necrosis and amputation of the affected digits in extreme cases. The classical biphasic color change during crisis should not be solely relied on to make the diagnosis of RP in black Africans.

## Abbreviations

ANA: Anti-nuclear antibodies
ASLO: Anti-streptolysin O antigen
CCBs: Calcium channel blockers
CREST: Calcinosis, Raynaud’s phenomenon, esophageal dysmotility, sclerodactyly, and telangiectasia
ESR: Erythrocyte sedimentation rate
PG E_2_: prostaglandin E_2_
RP: Raynaud’s phenomenon
